# Urinary Biomarkers in a Living Donor Kidney Transplantation Cohort—Predictive Value on Graft Function

**DOI:** 10.3390/ijms24065649

**Published:** 2023-03-15

**Authors:** G. J. Julia Huisman, Nora A. Spraakman, Jeroen V. Koomen, A. Marrit Talsma, Robert A. Pol, Stefan P. Berger, Henri G. D. Leuvenink, Michel M. R. F. Struys, Gertrude J. Nieuwenhuijs-Moeke

**Affiliations:** 1 University Medical Center Groningen, Department of Surgery, University of Groningen, Hanzeplein 1, 9713 GZ Groningen, The Netherlands; 2 University Medical Center Groningen, Department of Anesthesiology, University of Groningen, Hanzeplein 1, 9713 GZ Groningen, The Netherlands; 3 University Medical Centre Groningen, Department of Internal Medicine, Division of Nephrology, University of Groningen, Hanzeplein 1, 9713 GZ Groningen, The Netherlands; 4 Department of Basic and Applied Medical Sciences, Ghent University, 9000 Gent, Belgium

**Keywords:** kidney transplantation, urinary biomarkers, prediction models

## Abstract

Early non-invasive detection and prediction of graft function after kidney transplantation is essential since interventions might prevent further deterioration. The aim of this study was to analyze the dynamics and predictive value of four urinary biomarkers: kidney injury molecule-1 (KIM-1), heart-type fatty acid binding protein (H-FABP), N-acetyl-β-D-glucosaminidase (NAG), and neutrophil gelatinase-associated lipocalin (NGAL) in a living donor kidney transplantation (LDKT) cohort. Biomarkers were measured up to 9 days after the transplantation of 57 recipients participating in the VAPOR-1 trial. Dynamics of KIM-1, NAG, NGAL, and H-FABP significantly changed over the course of 9 days after transplantation. KIM-1 at day 1 and NAG at day 2 after transplantation were significant predictors for the estimated glomerular filtration rate (eGFR) at various timepoints after transplantation with a positive estimate (*p* < 0.05), whereas NGAL and NAG at day 1 after transplantation were negative significant predictors (*p* < 0.05). Multivariable analysis models for eGFR outcome improved after the addition of these biomarker levels. Several donor, recipient and transplantation factors significantly affected the baseline of urinary biomarkers. In conclusion, urinary biomarkers are of added value for the prediction of graft outcome, but influencing factors such as the timing of measurement and transplantation factors need to be considered.

## 1. Introduction

During the procedure of organ donation and transplantation, several potentially harmful processes occur, affecting the viability and quality of the kidney graft. Ischemia reperfusion injury (IRI) is inevitable and additional injury like brain death induced inflammation in the case of donation after brain death (DBD) donors, or prolonged warm ischemia in the case of donation after circulatory death (DCD) donors, may further harm the graft [1,2,3]. This is often clinically manifested in the early phase after transplantation as delayed graft function (DGF) or even primary non function (PNF), but may also make the graft prone to rejection or interstitial fibrosis and tubular atrophy (IFTA), reducing graft and patient survival [4,5,6]. New strategies to predict graft injury and/or dysfunction at an early stage might allow for early intervention and eventually improve the graft outcome [7]. Biomarkers, single or in combination, may be a helpful tool in this perspective and are used in animal experiments and clinical studies as a measure of injury. Various studies have suggested the use of non-invasive biomarkers for the prediction of early graft function [8,9]. Performance of these biomarkers, however, may vary between clinical syndromes or circumstances (e.g., acute kidney injury (AKI) or acute rejection) due to functions of the biomarker itself. This implies that the clinical utility of a biomarker needs to be carefully evaluated within the context it is used before it can be implemented in daily clinical care and prediction models [10,11,12].

Regarding kidney injury and graft outcome, several urinary biomarkers have been suggested including kidney injury molecule-1 (KIM-1), heart-type fatty acid binding protein (H-FABP), N-acetyl-β-D-glucosaminidase (NAG), and neutrophil gelatinase-associated Lipocalin (NGAL). These four biomarkers are upregulated, excreted, and/or released by the kidney at the time of injury like IRI, and could therefore be a proxy of the severity of damage [12]. These four biomarkers have been selected for various reasons. First of all, the biomarkers have different functions and origins in the nephron. KIM-1 has been indicated as a damage marker from the proximal tubule [13]. NAG is also released from the proximal tubule, but has shown both negative and positive relations with kidney outcome [14,15]. NGAL has also been indicated as a damage marker like KIM-1, but from the distal tubule [16]. H-FABP is predominantly present in the myocardium and to small mounts in the distal tubules of the kidney, and is therefore mainly evaluated in the context of cardiac surgery. However, recent studies have suggested the potential role of H-FABP as a biomarker following kidney transplantation [17]. Additionally, these biomarkers have been widely evaluated and used in animal and human studies, and were primary and/or secondary outcomes, thus stating the importance of these biomarkers [8]. However, research regarding the predictive value of KIM-1, H-FABP, NAG, and NGAL on graft outcome in kidney transplantation is scarce and the majority has focused on recipients of deceased kidney donors and AKI. During living donation, the duration of ischemia and the quality of the donor are substantially different to that of the deceased donors. In addition, studies often focus on one timepoint after transplantation and the possible dynamics of biomarkers over time are not considered. The current study therefore aimed to analyze the dynamics and predictive value of these urinary biomarkers for graft outcome measures in living donor kidney transplantation (LDKT).

## 2. Results

### 2.1. Baseline Characteristics

The baseline characteristics of the donors and recipients are summarized in Table 1. The course of the estimated glomerular filtration rate (eGFR) levels after transplantation is presented in Supplementary Materials, Figure S1.

### 2.2. Dynamics of Urinary Biomarkers in Recipients after Transplantation

**Figure 1:**
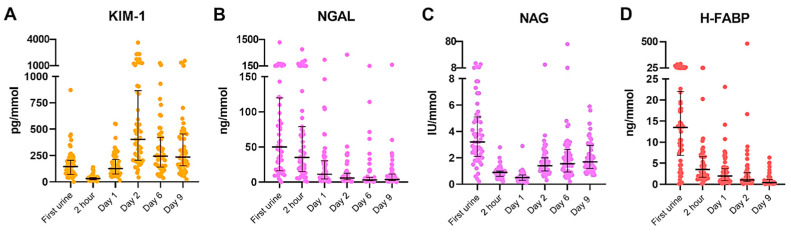
Post-transplantation urinary biomarkers, corrected for the urinary creatinine levels. Median urinary biomarker levels with interquartile range. (**A**) KIM-1, (**B**) NGAL, (**C**) NAG, (**D**) H-FABP. Abbreviations: KIM-1: kidney injury molecule-1, NGAL: neutrophil gelatinase-associated lipocalin, NAG: N-acetyl-β-D-glucosaminidase, H-FABP: heart-type fatty acid binding protein.

The dynamics of the urinary biomarkers are presented in Figure 1. KIM-1 showed a significant decrease from the first urine until two hours after transplantation (*p* < 0.001), followed by significant increases until day 2 (*p* < 0.001). Afterward, the KIM-1 levels decreased from day 2 until day 6 after transplantation (*p* < 0.001). The levels of NGAL significantly decreased after transplantation. We observed a significant decrease between 2 h and day 1 (*p* < 0.001), day 2 (*p* = 0.007) and day 6 (*p* < 0.001). The NAG levels showed a significant decrease between the first urine and day 1 after transplantation (*p* < 0.001), followed by a significant increase at day 2 (*p* < 0.001). The significant increase continued between day 6 and day 9 after transplantation (*p* = 0.043). Similar to NGAL, the levels of H-FABP decreased over 9 days. The H-FABP levels significantly decreased between first urine until day 1 after transplantation (*p* < 0.001), which continued between day 1 and day 2 (*p* = 0.002), and day 2 and day 9 (*p* < 0.001).

### 2.3. The Variability between Subjects of Urinary Biomarkers after Transplantation

In all models, time had a significant effect on the course of the urinary biomarkers (*p* < 0.001) and the addition of patient ID as a random effect improved the models. The best-fitting models are presented in Table 2. The intercept, representing an estimate of the baseline of a biomarker, of the KIM-1 model significantly increased if a sex mismatch was present (*p* = 0.0065) and with every min increase of second warm ischemia time (WIT, (*p* = 0.023)), but the univariable model for sex mismatch fitted the data best. Presence of a sex mismatch and an unrelated donor significantly increased the intercept (*p* = 0.0008 and *p* = 0.0228, respectively). Every year increase in the recipient age significantly increased the baseline of NAG activity with 0.0079 IU/mmol in a univariable model (*p* = 0.0213). The best-fitting model was represented by the addition of the sex mismatch. The baseline of the H-FABP model increased significantly by every minute increase of the cold ischemia time with 0.0065 ng/mmol (CIT, (*p* = 0.0189). The multivariable mixed models did not perform superior to the univariable mixed models.

### 2.4. The Additional Value of Urinary Biomarkers to Prediction Models for Renal Outcome

Univariable analysis of all the urinary biomarkers indicated several significant predictors for eGFR after transplantation, which are shown in Table 3. All univariable analysis results are included in Supplementary Tables S1–S4. KIM-1 at day 1 after transplantation was a significant positive predictor for 1-month (*p* = 0.010), 3-month (*p* = 0.034), 6-month (*p* = 0.025), 12-month (*p* = 0.008), and 24-month eGFR (*p* = 0.025). The NAG levels on day 1 were significant negative predictors for 1-month, (*p* = 0.017), 6-month (*p* = 0.006) and 24-month eGFR (*p* = 0.037). However, NAG on day 2 was a positive predictor for 6-month (*p* = 0.020) and 12-month eGFR (*p* = 0.022). NGAL on day 1 was a significant negative predictor for 1-month eGFR (*p* = 0.004). H-FBAP was not a significant predictor for the eGFR measurements. Changes between the first and last sample moment of all biomarkers were also implemented in regression models but did not show significant predictive value for renal outcome.

All significant predictors from the univariable regression analysis were implemented in a multivariable regression model, as shown in Table 4. The crude model consisted of significant donor, recipient, and transplantation variables. The addition of urinary biomarkers, alone or in combination, improved the model fit in all models, but the significance did not remain for all biomarkers. When analyzing the addition of separate biomarkers in the multivariable regression model, KIM-1 at day 1 after transplantation was a significant predictor for 1-month (*p* = 0.018), 3-month (*p* = 0.014), 6-month (*p* = 0.007), 12-month (*p* = 0.004), and 24 month eGFR (*p* = 0.016). The addition of NAG at day 1 and day 2 after transplantation improved the prediction model for 6-month eGFR (*p* = 0.050 and *p* = 0.006, respectively). NAG 2 days after transplantation was also a significant predictor for 12-month eGFR (*p* = 0.013). The levels of NGAL at day 1 after transplantation improved the prediction model for 1-month eGFR (*p* = 0.003).

## 3. Discussion

This post-hoc analysis shows that the levels of KIM-1, NAG, NGAL, and H-FABP significantly changed over the course of nine days after transplantation in our LDKT cohort, showing varied dynamic patterns. The patient, recipient, and transplantation characteristics explained the variability in the baseline between subjects. In addition, the predictive qualities differed per biomarker and between timepoints ranging from positive to negative prediction. This indicates that the timing and context of biomarker measurements are important when using these markers as an outcome measure in kidney injury research, since classically marked injury biomarkers may be involved in injury as well as repair mechanisms.

Our results are conflicting with known animal experiments and clinical trials, as we demonstrated a positive prediction of graft function with KIM-1 levels at day 1 after transplantation. KIM-1 is mainly viewed as a marker of kidney injury. Regarding kidney transplantation, most research evaluating KIM-1 has been performed in the recipients of deceased donor kidneys and showed an association of KIM-1 with suboptimal outcomes [18,19,20]. Most kidneys derived from deceased donors have already experienced an injurious event before retrieval, for instance, circulatory arrest or the systemic pro-inflammatory response due to brain death. In addition, these kidneys are exposed to a longer WIT and CIT. Furthermore, continued chronic expression of KIM-1 is associated with inflammation, the apoptosis of tubular cells, and fibrosis leading to inferior long-term outcome [20,21,22,23]. This may be the explanation that in studies in recipients of deceased donor kidneys, increased KIM-1 expression is related to worse outcome. The balance between kidney injury and levels of KIM-1 possibly defines the role of KIM-1 in injury or repair [24]. Moreover, the timing and endurance of KIM-1 expression seems to be the key to divergent KIM-1 signaling [25]. A possible explanation for this positive predictive function is related to the uptake of cellular debris, as it is crucial to clear the tubular lumen from cellular debris in order to prevent tubular obstruction and mediate the immune response [26,27,28,29,30]. As shown in our results, an early peak at day 1 is a positive predictor of graft function, but the continued increase at day 2 did not contribute to the prediction of graft outcome. Furthermore, KIM-1 was also involved in the repair of tissue integrity, and thus improved nephron function [31].

In contrast to KIM-1, NAG is a more disputable predictor with equivocal results. Urinary NAG activity at day 1 is a negative predictor for eGFR from 1 up to 24 months after transplantation. This is in line with previous research [14,32,33,34,35]. NAG is a lysosomal enzyme, present in many cells amongst which the proximal tubular cells of the nephron that release NAG by exocytosis or from the breakdown of cells. Therefore, an increased NAG activity could indicate renal injury, making it a potential biomarker for injury associated with kidney transplantation [11]. In contrast, NAG activity at day 2 after transplantation positively predicted eGFR after transplantation. NAG activity on this day might reflect regenerated tubular cells, showing a baseline lysosomal activity with higher levels indicating more regenerated cells. Kotanko et al. showed that a low urinary NAG activity between week two and four after transplantation was associated with poorer graft survival after four years compared to the high urinary NAG activity in this period [15].

In line with previous studies, NGAL was a negative predictor for outcome after transplantation outcome [16,36,37,38]. Release of NGAL by distal tubular epithelial cells increases the severity of kidney injury such as IRI associated with transplantation. NGAL was involved in various pathways including the regulation of apoptosis and induced renal tubule proliferation, which is a possible mechanism of protection during injury. Increased urinary NGAL levels after transplantation are thus potentially multifactorial as it is the result of both increased synthesis and decreased reabsorption by the tubules [39,40].

In this study, we demonstrated that the levels of H-FABP were the highest in the first produced urine after transplantation, followed by a significant decrease over time. Similarly, plasma H-FABP was found to be elevated after kidney transplantation and similar to our results, peaked immediately after reperfusion [17. A study by Schaub et al. demonstrated that the H-FABP levels, measured pre-operatively and up to three days post-operatively, were related to the development of AKI following cardiac surgery [41]. However, in our study, the H-FABP levels were not significantly related to the outcomes after transplantation. Our study showed that a longer CIT increased the intercept of the H-FABP model. Jochmans et al. demonstrated that the warm ischemia time significantly impacted the H-FABP [17].

Interestingly, the presence of a sex mismatch between the donor and recipient significantly increased the baseline of the multiple biomarker models. Several studies have shown that a sex mismatch has an impact on the graft. In a cohort of simultaneous pancreas–kidney transplantation, Coffman et al. demonstrated that organs derived from male donors had significantly higher graft survival rates compared to female donors [42]. To our knowledge, this is the first clinical study that demonstrates that sex mismatch impacts the dynamics of urinary biomarkers and potentially the graft early in the period after transplantation in a LDKT cohort. It is important to note that the effect of sex difference might be influenced by more than one patient-related factor [43]. Future studies that focus on the biological mechanisms and behavioral difference are needed to better interpret these results and explore the consequences in matching the donor and recipient in daily practice.

A few limitations of this study must be addressed. Our results only suggest associations and do not necessarily imply causality between the urinary biomarkers and eGFR levels or graft outcome. In addition, our sample size of 57 LDKT recipients was small and complications after transplantation like DGF were rare (*n* = 3). We therefore were not able to look at the association of the biomarker levels and graft dysfunction in terms of DGF. Therefore, further research is needed to determine the predictive value of these biomarkers on adverse graft outcomes in a larger study population. Another important limitation is the challenge in predicting the outcome after transplantation in the LDKT recipients. Fortunately, complications after transplantation were limited in number and graft function was relatively good. However, it may be beneficial to establish appropriate models for the prediction of short- and long-term graft outcome since not all recipients had a eGFR >50 mL/min/1.73 m^2^ after two years and acute rejection occurred in 16% of our study population. Furthermore, we needed to acknowledge the lack of multiple testing correction. This was an exploratory study, and we considered the analysis of every biomarker per timepoint with five repetitive eGFR measurements not to be a substantial risk for false positives. The fact that we sequentially measured the urinary biomarker levels is a major strength, which provides insight into the temporal character of urinary biomarkers in LDKT and the possible timeframes in which urinary biomarkers can be used as a biomarker. In addition, the fact that we used splint urine ensures that the biomarkers measured originate from the transplanted kidney only.

To conclude, the findings in our study advocate for a potential role of biomarkers in injury as well as repair mechanisms, changing their role from passive bystander released upon injury to an active role in regeneration. We show that the timing and context of biomarker measurements are important when using these markers as an outcome measure in kidney injury research. Further research is needed to determine the predictive functions in more detail and in different contexts of kidney injury. In the current study cohort of LDKT recipients, KIM-1, NAG, and NGAL appear to be potential biomarkers to predict kidney function up to two years after transplantation. When future research establishes the exact value of these biomarkers, the measurement of these urinary biomarkers after transplantation could facilitate the possibility to closely monitor renal function in a non-invasive manner and adjust treatments, thereby improving the patient outcomes.

## 4. Materials and Methods

### 4.1. Study Design

This study is a post-hoc analysis of the VAPOR-1 (Volatile Anesthetic Protection of Renal Transplants-1) trial, a prospective randomized controlled trial on the effect of two different anesthetic agents (propofol vs. sevoflurane) on renal outcome in LDKT. The VAPOR-1 trial was approved by the local Institutional Review Board (METc 2009/334), registered with ClinicalTrials.gov (NCT01248871) and conducted at the University Medical Center of Groningen between September 2010 and October 2014. Details of the study have previously been published [44]. A total of 60 donor–recipient couples (120 patients in total) met the inclusion criteria, gave written informed consent, and were randomly assigned to one of the following groups: PROP, propofol for the donor and recipient; SEVO, sevoflurane for the donor and recipient; and PROSE, propofol for the donor and sevoflurane for the recipient. Three couples were excluded from the primary analysis due to violation of the surgical or immunosuppressive protocol, leaving 57 couples (57 donors and 57 recipients) for analysis. For this analysis, the recipients were pooled in one group.

### 4.2. Outcome Measures

The primary outcome was to study the release of four urinary biomarkers: KIM-1, NGAL, NAG, and HFABP in the period after transplantation. These biomarkers were chosen as they either represent different injury mechanisms or have a different origin in the kidney. Secondary outcomes were predictive values of urinary biomarkers to eGFR, the occurrence of DGF, acute rejection episodes, graft loss, and patient mortality. In recipients, eGFR was calculated with the use of the CKD-EPI formula at month 1-, 3-, 6-, 12-, and 24-months after transplantation.

### 4.3. Timepoints

**Figure 2:**
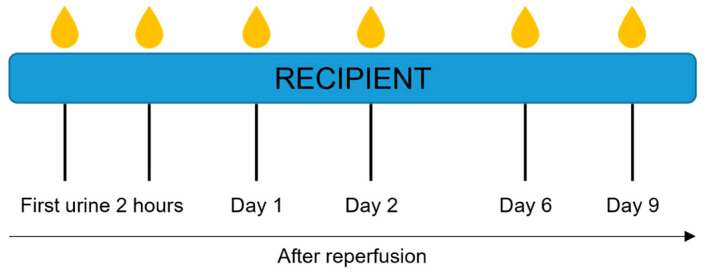
Timeline sample collection in the recipient.

Urine samples were taken at standardized time points. Sample points in recipients were first urine produced upon reperfusion, 2 h post-operative and on days 1, 2, 6, and 9 after transplantation. Urinary samples were collected from a splint (representing urine solely from the transplanted kidney). H-FABP was not measured on day 6. Timepoints are depicted in Figure 2.

### 4.4. Measurement of Urinary Biomarkers

The urinary KIM-1, NGAL, and H-FABP levels were measured with the duoset enzyme-linked immunosorbent assay (ELISA; R&D systems, Minneapolis, MN, USA). NAG was measured by the modified enzyme assay using p-nitrophenyl-N-acetyl-β-D-glucosaminidase as the substrate. Urinary creatinine was determined on a Roche Modular chemistry analyzer (Roche Diagnostics, Indianapolis, IN, USA). KIM-1, H-FABP, and NAG were measured for the VAPOR-1 study. For this post-hoc analysis, we additionally measured NGAL.

### 4.5. Statistical Analysis

Statistical analysis was performed in SPSS version 28 (IBM Corp, Armonk, NY, USA), GraphPad Prism version 8.0.1. (GraphPad software, Inc., La Jolla, CA, USA), and R Studio (v.1.4.1106, PBC, Boston, MA, USA). It was tested whether the continuous variables followed a normal distribution with the use of the Shapiro–Wilk normality test and visualization by the normal probability (Q–Q) plots. Data were corrected for extreme outliers (>2 SD). Descriptive statistics were presented as the mean (±SD) for the normally distributed variables, median (interquartile range (IQR)) in the case of non-normal distributions, or proportions n with corresponding percentages (%) for the categorical variables. Before statistical testing, we log transformed the urinary biomarker to better approximate a normal distribution. Statistical significance was set at a *p*-value ≤ 0.05 for all comparisons.

First, we analyzed the dynamics and the variability between the subjects of the urinary biomarkers. For the dependent variables, a paired *T*-test or Wilcoxon signed rank test was performed. Linear mixed-effects models were used to characterize and explain the variability of the urinary biomarkers over time, where time was entered as a fixed effect. The time effect was evaluated assuming a linear relationship, but nonlinear trends were also evaluated using polynomial functions. Estimates of the covariates are presented as b with standard error (SE). Patient identification was entered as a random effect. Donor, recipient, and transplantation characteristics (based on literature) were evaluated on the fixed effects, representing the baseline and parameters describing changes over time, on their ability to explain the variability in urinary markers. Combinations of significant variables in the univariable models were tested in the multivariable models to study whether a combination of characteristics resulted in additional explanation of the variability in the biomarkers. The best-fitted models are presented based on the Akaike information criteria (AIC) and significance of the model parameters.

Next, we evaluated the predictive value of the urinary biomarkers for the transplantation outcomes. Univariable linear regression analyses were performed to analyze the urinary biomarkers as predictive variables in relation to the renal outcome parameters. Significant predictive variables in the univariable models were added to a multivariable regression model to assess the improvement in the prediction, based on the adjusted R-square.

## Figures and Tables

**Table 1:** The baseline characteristics of the donors and recipients.

**Donor**	***n* = 57**
Age [y]	52.3 (±11.0)
Male [*n*(%)]	26 (45.6%)
BMI [kg/m^2^]	27 (±3.2)
Active smokers [*n*(%)]	16 (28.1%)
Cardiovascular comorbidity [*n*(%)]	17 (29.8%)
Medication use [*n*(%)]	
*Antihypertensive therapy*	15 (26.3%)
Statins	7 (12.3%)
PPI’s	9 (15.8%)
Pre-donation mGFR [mL/min]	116 (97–134)
**Recipient**	***n* = 57**
Age [y]	51.2 (45.0–58.5)
Male [*n*(%)]	27 (47.4%)
BMI [kg/m^2^]	25.4 (22.5–28.3)
Cardiovascular comorbidity [*n*(%)]	39(68.4%)
Medication use [*n*(%)]	
*Antihypertensive therapy*	52 (91.2%)
*Phosphate binders*	32 (56.1%)
*Statins*	28 (49.1%)
Unrelated donor [*n*(%)]	29 (50.9%)
Pre-emptive transplantation [*n*(%)]	28 (49.1%)
Re-transplantation [*n*(%)]	7 (12.3%)
≥3 HLA mismatches [*n*(%)]	35 (61.4%)
Positive PRA [*n*(%)]	7 (12.3%)
Ischemia times [min]	
*WIT 1*	4 (3–4)
*CIT*	175.5 (156.0–187.0)
*WIT 2*	43.0 (± 7.3)
**Kidney and Patient Outcomes**	***n* = 57**
DGF [*n*(%)]	3 (5.4%)
eGFR 1 month post transplantation [mL/min/1.73 m^2^]	50.8 (±14.9)
eGFR 3 months post transplantation [mL/min/1.73 m^2^]	49.6 (38.8–58.2)
eGFR 6 months post transplantation [mL/min/1.73 m^2^]	50.4 (38.8–61.1)
eGFR 12 months post transplantation [mL/min/1.73 m^2^]	50.2 (±14.2)
eGFR 24 months post transplantation [mL/min/1.73 m^2^]	51.4 (±17.6)
Acute rejection 2 years [*n*(%)]	9 (16.1%)
Graft loss [*n*(%)]	2 (3.5%)
Mortality [*n*(%)]	1 (1.8%)

Data given as mean (± SD), median (IQR) or *n*(%). Abbreviations: *n*: number in group; BMI: body mass index; PPI’s: proton pump inhibitors; HLA: human leukocytes antigens; PRA: panel specific antibodies ≥15%; WIT1: warm ischemia time 1; CIT: cold ischemia time; WIT2: warm ischemia time 2, DGF: delayed graft function, mGFR: measured glomerular filtration rate, eGFR: estimated glomerular filtration rate.

**Table 2:** The univariable mixed-effects models of the urinary biomarkers.

Urinary Biomarker	Variable (Effect)	B (Unit)	SE of b	*p*-Value
KIM-1	Sex mismatch (intercept)	0.39 (male/female)	0.14	0.0065
	WIT2 (intercept)	0.030 (min)	0.009	0.0023
NAG	Sex mismatch (intercept)	0.28 (yes/no)	0.08	0.0008
	Unrelated donor (intercept)	0.19 (no/yes)	0.08	0.0228
	Recipient age (intercept)	0.0079 (years)	0.003	0.0213
H-FABP	CIT (intercept)	0.0065 (min)	0.003	0.0189

Best-fitted models are presented with an estimate of the fixed effects (b with SE). b is expressed with additional information about the unit: either as a categorical variable or as a continuous variable (with type of unit). Abbreviations: KIM-1: kidney injury molecule-1; NAG: N-acetyl-β-D-glucosaminidase; HFABP: heart-type fatty acid binding protein; WIT2: eGFR: estimated glomerular filtration rate; CIT: cold ischemia time.

**Table 3:** The univariable regressions of the urinary biomarkers.

**1-Month eGFR**		
*Variable*	*p-Value*	*Estimate (95% CI)*
KIM-1 day 1	0.010	7.71 (1.96–13.46)
NAG day 1	0.017	−8.26 (−14.99–−1.53)
NGAL day 1	0.004	−4.55 (−7.58–−1.51)
**3-Month eGFR**		
*Variable*	*p-Value*	*Estimate (95% CI)*
KIM-1 day 1	0.034	6.80 (0.54–13.06)
**6-Month eGFR**		
*Variable*	*p-Value*	*Estimate (95% CI)*
KIM-1 day 1	0.025	6.57 (0.86–12.27)
NAG day 1	0.006	−9.65 (−16.37–−2.94)
NAG day 2	0.020	8.89 (1.47–16.31)
**12-Month eGFR**		
*Variable*	*p-Value*	*Estimate (95% CI)*
KIM-1 day 1	0.008	7.33 (1.99–12.66)
NAG day 2	0.022	8.42 (1.24–15.60)
**24-Month eGFR**		
*Variable*	*p-Value*	*Estimate (95% CI)*
KIM-1 day 1	0.025	7.82 (1.00–14.63)
NAG day 1	0.037	8.87 (−17.20–−0.55)

Data given as *p*-values and estimates (b with confidence interval (CI)). Abbreviations: KIM-1: kidney injury molecule-1; NGAL: neutrophil gelatinase-associated lipocalin; NAG: N-acetyl-β-D-glucosaminidase; HFABP: heart-type fatty acid binding protein.

**Table 4:** Multivariable regressions and the addition of urinary biomarkers to the crude model.

**1-Month eGFR**				
	*Variable*	*Adjusted R^2^*	*p-Value*	*Estimate (95% CI)*
Crude model	Donor sex	0.255	0.009	−9.79 (−16.98–2.60)
	Donor age		0.007	−0.46 (−0.80–−0.13)
Addition of biomarker	KIM day 1	0.323	0.018	6.39 (1.16–11.62)
	NAG day 1	0.259	0.089	−5.83 (−12.56–0.93)
	NGAL day 1	0.259	0.003	−4.39 (−7.16–1.62)
Addition of===>1 biomarker	KIM day 1	0.396	0.001	8.70 (3.63–13.77)
	NAG day 1		0.117	−4.64 (−10.50–1.12)
	NGAL day 1		0.125	−2.20 (−5.03–0.63)
**3-Month eGFR**				
	*Variable*	*Adjusted R^2^*	*p-Value*	*Estimate (95% CI)*
Crude model	Donor age	0.203	<0.001	−0.68 (−1.04–−0.33)
Addition of biomarker	KIM-1 day 1	0.272	0.014	7.06 (1.52–12.60)
**6-Month eGFR**				
	*Variable*	*Adjusted R^2^*	*p-Value*	*Estimate (95% CI)*
Crude model	Donor age	0.246	<0.001	−0.70 (−1.02–−0.38)
Addition of biomarker	KIM-1 day 1	0.317	0.007	6.82 (1.91–11.73)
	NAG day 1	0.276	0.050	−6.42 (−12.85–0.01)
	NAG day 2	0.319	0.006	9.18 (2.79–15.57)
Addition of >1 biomarker	KIM-1 day 1	0.373	0.090	4.59 (−0.74–9.91)
	NAG day 1		0.056	−5.83 (−11.80–0.15)
	NAG day 2		0.037	7.44 (0.48–14.40)
**12-Month eGFR**				
	*Variable*	*Adjusted R^2^*	*p-Value*	*Estimate (95% CI)*
Crude model	Donor age	0.120	0.005	−0.48 (−0.82–−0.15)
Addition of biomarker	KIM-1 day 1	0.240	0.004	7.51 (2.56–12.45)
	NAG day 2	0.189	0.013	8.62 (1.89–15.36)
Addition of >1 biomarker	KIM-1 day 1	0.264	0.035	5.64 (0.40–10.88)
	NAG day 2		0.045	7.31 (0.17–14.45)
**24-Month eGFR**				
	*Variable*	*Adjusted R^2^*	*p-Value*	*Estimate (95% CI)*
Crude model	Donor age	0.106	0.009	−0.57 (−0.98–−0.15)
Addition of biomarker	KIM-1 day 1	0.182	0.016	8.00 (1.57–14.43)
	NAG day 1	0.118	0.137	−6.40 (−14.91–2.10)
Addition of >1 biomarker	KIM-1 day 1	0.200	0.019	8.12 (1.37–14.86)
	NAG day 1		0.182	−5.48 (−13.62–2.66)

Data given as adjusted R-square (R^2^), *p*-values and estimates (b with confidence interval (CI)). Abbreviations: KIM-1: kidney injury molecule-1; NGAL: neutrophil gelatinase-associated lipocalin; NAG: N-acetyl-β-D-glucosaminidase; HFABP: heart-type fatty acid binding protein.

## Data Availability

The data that support the findings of this study are available from the corresponding author upon reasonable request.

## Supplementary Materials

The following supporting information can be downloaded at: https://www.mdpi.com/article/10.3390/ijms24065649/s1.

## References

[1] Ponticelli C. (2014). Ischaemia-reperfusion injury: A major protagonist in kidney transplantation. Nephrol. Dial. Transplant..

[2] Cooper J.E., Wiseman A.C. (2013). Acute kidney injury in kidney transplantation. Curr. Opin. Nephrol. Hypertens..

[3] Salvadori M., Rosso G., Bertoni E. (2015). Update on ischemia-reperfusion injury in kidney transplantation: Pathogenesis and treatment. World. J. Transplant..

[4] Yarlagadda S.G., Coca S.G., Formica R.N., Poggio E.D., Parikh C.R. (2009). Association between delayed graft function and allograft and patient survival: A systematic review and meta-analysis. Nephrol. Dial. Transplant..

[5] Nieuwenhuijs-Moeke G.J., Pischke S.E., Berger S.P., Sanders J.S.F., Pol R.A., Struys M., Ploeg R.J., Leuvenink H.G.D. (2020). Ischemia and Reperfusion Injury in Kidney Transplantation: Relevant Mechanisms in Injury and Repair. J. Clin. Med..

[6] Koo T.Y., Jeong J.C., Lee Y., Ko K.P., Lee K.B., Lee S., Park S.J., Park J.B., Han M., Lim H.J. (2016). Pre-transplant Evaluation of Donor Urinary Biomarkers can Predict Reduced Graft Function After Deceased Donor Kidney Transplantation. Medicine.

[7] Malyszko J., Lukaszyk E., Glowinska I., Durlik M. (2015). Biomarkers of delayed graft function as a form of acute kidney injury in kidney transplantation. Sci. Rep..

[8] Halawa A. (2011). The early diagnosis of acute renal graft dysfunction: A challenge we face. The role of novel biomarkers. Ann. Transplant..

[9] Kers J., Peters-Sengers H., Heemskerk M.B.A., Berger S.P., Betjes M.G.H., van Zuilen A.D., Hilbrands L.B., de Fijter J.W., Nurmohamed A.S., Christiaans M.H. (2020). Prediction models for delayed graft function: External validation on The Dutch Prospective Renal Transplantation Registry. Nephrol. Dial. Transplant..

[10] Vanmassenhove J., Vanholder R., Nagler E., Van Biesen W. (2013). Urinary and serum biomarkers for the diagnosis of acute kidney injury: An in-depth review of the literature. Nephrol. Dial. Transplant..

[11] Beker B.M., Corleto M.G., Fieiras C., Musso C.G. (2018). Novel acute kidney injury biomarkers: Their characteristics, utility and concerns. Int. Urol. Nephrol..

[12] Obermuller N., Geiger H., Weipert C., Urbschat A. (2014). Current developments in early diagnosis of acute kidney injury. Int. Urol. Nephrol..

[13] Lin Q., Chen Y., Lv J., Zhang H., Tang J., Gunaratnam L., Li X., Yang L. (2014). Kidney injury molecule-1 expression in IgA nephropathy and its correlation with hypoxia and tubulointerstitial inflammation. Am. J. Physiol. Renal. Physiol..

[14] Kwiatkowska E., Domanski L., Bober J., Kloda K., Safranow K., Szymanska-Pasternak J., Romanowski M., Sulecka A., Pawlik A., Ciechanowski K. (2014). N-acetyl-beta-glucosaminidase urine activity as a marker of early proximal tubule damage and a predictor of the long-term function of the transplanted kidneys. Acta Biochim. Pol..

[15] Kotanko P., Margreiter R., Pfaller W. (1996). Reduced renal allograft survival is related to low urinary N-acetyl-beta-D-glucosaminidase excretion during the first posttransplant month. Transplantation.

[16] Cappuccilli M., Capelli I., Comai G., Cianciolo G., La Manna G. (2018). Neutrophil Gelatinase-Associated Lipocalin as a Biomarker of Allograft Function After Renal Transplantation: Evaluation of the Current Status and Future Insights. Artif. Organs.

[17] Jochmans I., Lerut E., van Pelt J., Monbaliu D., Pirenne J. (2011). Circulating AST, H-FABP, and NGAL are early and accurate biomarkers of graft injury and dysfunction in a preclinical model of kidney transplantation. Ann. Surg..

[18] Bank J.R., van der Pol P., Vreeken D., Monge-Chaubo C., Bajema I.M., Schlagwein N., van Gijlswijk D.J., van der Kooij S.W., Reinders M.E.J., de Fijter J.W. (2017). Kidney injury molecule-1 staining in renal allograft biopsies 10 days after transplantation is inversely correlated with functioning proximal tubular epithelial cells. Nephrol. Dial. Transplant..

[19] Schroppel B., Kruger B., Walsh L., Yeung M., Harris S., Garrison K., Himmelfarb J., Lerner S.M., Bromberg J.S., Zhang P.L. (2010). Tubular expression of KIM-1 does not predict delayed function after transplantation. J. Am. Soc. Nephrol..

[20] Van Timmeren M.M., Vaidya V.S., van Ree R.M., Oterdoom L.H., de Vries A.P., Gans R.O., van Goor H., Stegeman C.A., Bonventre J.V., Bakker S.J. (2007). High urinary excretion of kidney injury molecule-1 is an independent predictor of graft loss in renal transplant recipients. Transplantation.

[21] Zhang P.L., Rothblum L.I., Han W.K., Blasick T.M., Potdar S., Bonventre J.V. (2008). Kidney injury molecule-1 expression in transplant biopsies is a sensitive measure of cell injury. Kidney Int..

[22] Tian L., Shao X., Xie Y., Wang Q., Che X., Zhang M., Xu W., Xu Y., Mou S., Ni Z. (2017). Kidney Injury Molecule-1 is Elevated in Nephropathy and Mediates Macrophage Activation via the Mapk Signalling Pathway. Cell Physiol. Biochem..

[23] Humphreys B.D., Xu F., Sabbisetti V., Grgic I., Movahedi Naini S., Wang N., Chen G., Xiao S., Patel D., Henderson J.M. (2013). Chronic epithelial kidney injury molecule-1 expression causes murine kidney fibrosis. J. Clin. Investig..

[24] Gandhi R., Yi J., Ha J., Shi H., Ismail O., Nathoo S., Bonventre J.V., Zhang X., Gunaratnam L. (2014). Accelerated receptor shedding inhibits kidney injury molecule-1 (KIM-1)-mediated efferocytosis. Am. J. Physiol. Renal. Physiol..

[25] Brooks C.R., Bonventre J.V. (2015). KIM-1/TIM-1 in proximal tubular cell immune response. Oncotarget.

[26] Ichimura T., Asseldonk E.J., Humphreys B.D., Gunaratnam L., Duffield J.S., Bonventre J.V. (2008). Kidney injury molecule-1 is a phosphatidylserine receptor that confers a phagocytic phenotype on epithelial cells. J. Clin. Investig..

[27] Brooks C.R., Yeung M.Y., Brooks Y.S., Chen H., Ichimura T., Henderson J.M., Bonventre J.V. (2015). KIM-1-/TIM-1-mediated phagocytosis links ATG5-/ULK1-dependent clearance of apoptotic cells to antigen presentation. EMBO J..

[28] Bonventre J.V., Yang L. (2010). Kidney injury molecule-1. Curr. Opin. Crit. Care.

[29] Yang L., Brooks C.R., Xiao S., Sabbisetti V., Yeung M.Y., Hsiao L.L., Ichimura T., Kuchroo V., Bonventre J.V. (2015). KIM-1-mediated phagocytosis reduces acute injury to the kidney. J. Clin. Investig..

[30] Lee J.Y., Ismail O.Z., Zhang X., Haig A., Lian D., Gunaratnam L. (2018). Donor kidney injury molecule-1 promotes graft recovery by regulating systemic necroinflammation. Am. J. Transplant..

[31] Zhang Z., Cai C.X. (2016). Kidney injury molecule-1 (KIM-1) mediates renal epithelial cell repair via ERK MAPK signaling pathway. Mol. Cell Biochem..

[32] Kuzniar J., Marchewka Z., Krasnowski R., Boratynska M., Dlugosz A., Klinger M. (2006). Enzymuria and low molecular weight protein excretion as the differentiating marker of complications in the early post kidney transplantation period. Int. Urol. Nephrol..

[33] Kind P.R. (1982). N-Acetyl-beta-D-glucosaminidase in urine of patients with renal disease, and after renal transplants and surgery. Clin. Chim. Acta.

[34] De Muro P., Faedda R., Masala A., Lepedda A.J., Zinellu E., Ciccarese M., Cossu M., Pala P.G., Satta R.P., Formato M. (2013). Kidney post-transplant monitoring of urinary glycosaminoglycans/proteoglycans and monokine induced by IFN-gamma (MIG). Clin. Exp. Med..

[35] Nauta F.L., Bakker S.J., van Oeveren W., Navis G., van der Heide J.J., van Goor H., de Jong P.E., Gansevoort R.T. (2011). Albuminuria, proteinuria, and novel urine biomarkers as predictors of long-term allograft outcomes in kidney transplant recipients. Am. J. Kidney Dis..

[36] Devarajan P. (2008). Neutrophil gelatinase-associated lipocalin (NGAL): A new marker of kidney disease. Scand. J. Clin. Lab. Investig..

[37] Qurashi S., Ghamdi G., Jaradat M., Tamim H., Aljumah A., Tamimi W., Al Dawood A., Binsalih S., Al Sayyari A. (2014). Urinary neutrophil gelatinase-associated lipocalin and the occurrence of delayed graft function after kidney transplant. Exp. Clin. Transplant..

[38] Parikh C.R., Jani A., Mishra J., Ma Q., Kelly C., Barasch J., Edelstein C.L., Devarajan P. (2006). Urine NGAL and IL-18 are predictive biomarkers for delayed graft function following kidney transplantation. Am. J. Transplant..

[39] Ramirez-Sandoval J.C., Herrington W., Morales-Buenrostro L.E. (2015). Neutrophil gelatinase-associated lipocalin in kidney transplantation: A review. Transplant. Rev..

[40] Buemi A., Musuamba F., Frederic S., Douhet A., De Meyer M., De Pauw L., Darius T., Kanaan N., Wallemacq P., Mourad M. (2014). Is plasma and urine neutrophil gelatinase-associated lipocalin (NGAL) determination in donors and recipients predictive of renal function after kidney transplantation?. Clin. Biochem..

[41] Schaub J.A., Garg A.X., Coca S.G., Testani J.M., Shlipak M.G., Eikelboom J., Kavsak P., McArthur E., Shortt C., Whitlock R. (2015). Perioperative heart-type fatty acid binding protein is associated with acute kidney injury after cardiac surgery. Kidney Int..

[42] Coffman D.J., Jay C.L., Sharda B., Garner M., Farney A.C., Orlando G., Reeves-Daniel A., Mena-Gutierrez A., Sakhovskaya N., Stratta R. (2023). Influence of donor and recipient sex on outcomes following simultaneous pancreas-kidney transplantation in the new millennium: Single-center experience and review of the literature. Clin. Transplant..

[43] Lepeytre F., Dahhou M., Zhang X., Boucquemont J., Sapir-Pichhadze R., Cardinal H., Foster B.J. (2017). Association of Sex with Risk of Kidney Graft Failure Differs by Age. J. Am. Soc. Nephrol..

[44] Nieuwenhuijs-Moeke G.J., Nieuwenhuijs V.B., Seelen M.A.J., Berger S.P., van den Heuvel M.C., Burgerhof J.G.M., Ottens P.J., Ploeg R.J., Leuvenink H.G.D., Struys M. (2017). Propofol-based anaesthesia versus sevoflurane-based anaesthesia for living donor kidney transplantation: Results of the VAPOR-1 randomized controlled trial. Br. J. Anaesth..

