# Supplementary material for: Urinary Biomarkers in a Living Donor Kidney Transplantation Cohort—Predictive Value on Graft Function
Source: Int J Mol Sci. 2023 Mar 15;24(6):5649. doi: 10.3390/ijms24065649 (PMC10054503; doi:10.3390/ijms24065649)
Supplement: Supplementary file 1 [file ijms-24-05649-s001.zip › ijms-2249645-supplementary.pdf]

SUPPLEMENTARY TABLES

Table S1. Univariable analysis of KIM-1 levels.

	GFR 1 month	GFR 3 months	GFR 6 months	GFR 12 months	GFR 24 months
KIM first urine	B=3.74 (-2.55 – 10.03) P=0.238	B=4.59 (-1.97 - 11.14) P=0.166	B=2.05 (-4.17 – 8.27) P=0.511	B=2.86 (-3.20 – 8.92) P=0.348	B=1.11 (-6.57 – 8.79) P=0.772
KIM 2 hours	B=1.49 (-6.70 – 9.67) P=0.717	B=1.07 (-7.15 – 9.29) P=0.795	B=2.09 (-5.79 – 9.97) P=0.597	B=0.09 (-7.64 – 7.82) P=0.981	B=2.61 (-7.38–12.60) P=0.602
KIM 1 day	**B=7.17 (1.96 – 13.46) P=0.010**	**B=6.80 (0.54 -13.06) P=0.034**	**B=6.56 (0.86 – 12.27) P=0.025**	**B=7.33 (1.99 – 12.66) P=0.008**	**B=7.82 (1.00 – 14.63) P=0.025**
KIM 2 days	B=4.00 (-0.73 – 8.73) P=0.096	B=2.41 (-2.66 – 7.47) P=0.345	B=3.08 (-1.66 – 7.81) P=0.198	B=0.27 (-4.36 – 4.90) P=0.907	B=1.07 (-4.63 – 6.76) P=0.708
KIM 6 days	B=2.13 (-3.40 – 7.66) P=0.441	B=3.01 (-2.63 – 8.65) P=0.288	B=3.81 (-1.30 – 8.91) P=0.140	B=2.11 (-2.93 – 7.14) P=0.404	B=1.57 (-4.72 – 7.86) P=0.617
KIM 9 days	B=3.92 (-1.53 – 9.37) P=0.154	B=4.44 (-0.80 – 9.67) P=0.095	B=3.26 (-1.93 – 8.46) P=0.213	B=2.64 (-2.51 – 7.80) P=0.308	B=4.33 (-1.9 – 10.63) P=0.173

Data given as P-values and estimates (b with confidence interval (CI)). Abbreviations: KIM-1: kidney injury molecule-1.

Table S2. Univariable analysis of NAG levels.

	GFR 1 month	GFR 3 months	GFR 6 months	GFR 12 months	GFR 24 months
NAG first urine	B=2.30 (-4.13 – 8.73) P=0.476	B=4.75 (-1.89 – 11.39) P=0.157	B=2.34 (-3.95 – 8.63) P=0.458	B=5.28 (-0.67 – 11.24) P=0.082	B=6.21 (-1.34 – 13.76) P=0.104
NAG 2 hours	B=-6.38 (-16.11 – 3.34) P=0.194	B=3.36 (-7.15 – 13.86) P=0.524	B=-0.81 (-10.62 – 9.01) P=0.870	B=0.73 (-8.72 – 10.18) P=0.878	B=2.35 (-9.36 – 14.06) P=0.689
NAG 1 day	**B=-8.26 (-14.99 - -1.53) P=0.017**	B=-5.14 (-12.94 – 2.65) P=0.191	**B=-9.65 (-16.37 – -2.94) P=0.006**	B=-4.50 (-11.21 – 2.21) P=0.184	**B=-8.87 (-17.20 – -0.55) P=0.037**
NAG 2 days	B=6.26 (-1.44 – 13.95) P=0.109	B=7.25 (-0.94 – 15.44) P=0.081	**B=8.89 (1.47 – 16.31) P=0.020**	**B=8.42 (1.24 – 15.57) P=0.022**	B=6.79 (-2.49 – 16.06) P=0.148
NAG 6 days	B=-0.79 (-7.92 – 6.34) P=0.825	B=1.46 (-6.14 – 9.05) P=0.702	B=2.17 (-4.82 – 9.17) P=0.535	B=1.24 (-5.57 – 8.05) P=0.717	B=-2.10 (-10.60 – 6.40) P=0.622
NAG 9 days	B=-1.91 (-9.77 – 5.96) P=0.628	B=-0.37 (-7.84 – 8.57) P=0.929	B=-1.25 (-8.99 – 6.49) P=0.747	B=0.01 (-7.44 – 7.46) P=0.998	B=-0.25 (-9.55 – 9.05) P=0.958

Data given as P-values and estimates (b with confidence interval (CI)). Abbreviations: NAG: N-acetyl-β-D-glucosaminidase.

Table S3. Univariable analysis of NGAL levels.

	GFR 1 month	GFR 3 months	GFR 6 months	GFR 12 months	GFR 24 months
NGAL first urine	B=0.35(-2.80 – 3.49) P=0.825	B=0.66 (-2.64 – 3.97) P=0.688	B=0.88 (-2.17 – 3.93) P=0.564	B=0.03 (-2.91 – 2.97) P=0.983	B=1.40 (-2.26 – 5.05) P=0.446
NGAL 2 hours	B=-1.12 (-4.62 – 2.39) P=0.410	B=-1.01 (-4.81 – 2.80) P=0.597	B=-0.67 (-4.30 – 2.97) P=0.714	B=-1.43 (-4.81 – 1.95) P=0.399	B=1.01 (-3.39 - 5.42) P=0.645
NGAL 1 day	**B=-4.55 (-7.58 – -1.51) P=0.004**	B=-2.20 (-5.69 – 1.28) P=0.209	B=-1.92 (-5.10 – 1.26) P=0.231	B=-1.60 (-4.63 – 1.43) P=0.294	B=-2.43 (-6.23 – 1.37) P=0.204
NGAL 2 days	B=-1.77 (-7.33 – 3.79) P=0.552	B=-0.89 (-8.35 – 6.58) P=0.811	B=-1.24 (-8.12 – 5.63) P=0.715	B=0.00[5] (-6.02 – 6.02) P=0.999	B=-1.60 (-8.58 – 5.39) P=0.645
NGAL 6 days	B=-2.17 (-7.46 – 3.12) P=0.412	B=-2.79 (-8.99 – 3.42) P=0.370	B=-2.29 (-8.01 – 3.44) P=0.425	B=-2.31 (-7.49 – 2.88) P=0.374	B=-0.43 (-7.30 – 6.44) P=0.900
NGAL 9 days	B=-2.27 (-6.21 – 1.67) P=0.251	B=-2.49 (-6.86 – 1.88) P=0.256	B=-2.62 (-6.65 – 1.42) P=0.199	B=-1.15 (-5.01 – 2.70) P=0.550	B=1.29 (-3.69 – 6.27) P=0.604

Data given as P-values and estimates (b with confidence interval (CI)). Abbreviations: NGAL: Neutrophil gelatinase-associated lipocalin.

Table S4. Univariable analysis of H-FABP levels.

	GFR 1 month	GFR 3 months	GFR 6 months	GFR 12 months	GFR 24 months
H-FABP first urine	B=1.41 (-2.69 – 5.50)<br>P=0.492	B=0.17 (-4.18 – 4.52)<br>P=0.938	B=-0.11 (-4.12 – 3.90)<br>P=0.957	B=0.09 (-3.86 – 4.04)<br>P=0.964	B=1.13 (-3.80 – 6.07)<br>P=0.646
H-FABP 2 hours	B=2.93 (-1.67 – 7.54)<br>P=0.207	B=0.402 (-0.91 – 8.95)<br>P=0.107	B=3.82 (-0.75 – 8.40)<br>P=0.099	B=4.29 (-0.09 – 8.66)<br>P=0.055	B=2.24 (-4.08 – 8.56)<br>P=0.479
H-FABP 1 day	B=0.14 (-4.47 – 4.76)<br>P=0.951	B=-0.01 (-5.16 – 5.14)<br>P=0.996	B=-0.59 (-4.10 – 5.27)<br>P=0.803	B=-0.27 (-4.70 – 4.16)<br>P=0.902	B=1.77 (-3.89 – 7.42)<br>P=0.532
H-FABP 2 days	B=-4.05 (-8.49 – 0.39)<br>P=0.073	B=-2.18 (-7.01 – 2.70)<br>P=0.374	B=-1.39 (-5.97 – 3.18)<br>P=0.543	B=-0.86 (-5.29 – 3.56)<br>P=0.697	B=-1.43 (-7.17 – 4.31)<br>P=0.618
H-FABP 9 days	B=-3.48 (-8.44 – 1.48)<br>P=0.165	B=-2.55 (-7.87 – 2.78)<br>P=0.341	B=-0.94 (-5.89 – 4.02)<br>P=0.706	B=-0.63 (-5.44 – 4.17)<br>P=0.793	B=-3.34 (-9.30 – 2.62)<br>P=0.265

Data given as P-values and estimates (b with confidence interval (CI)). Abbreviations: HFABP: heart-type fatty acid binding protein.

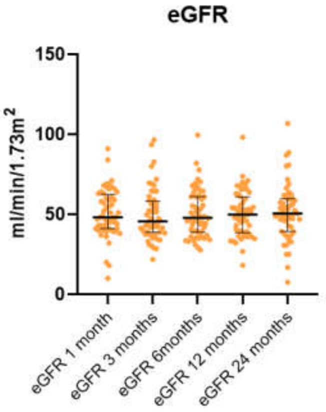

Figure S1. Post-transplantation eGFR levels. Median eGFR levels with interquartile range. Abbreviations: eGFR: estimated glomerular filtration rate.
